# Orderliness/Disorderliness Is Mentally Associated With Construal Level and Psychological Distance

**DOI:** 10.3389/fpsyg.2019.02521

**Published:** 2019-11-20

**Authors:** Kaiyun Li, Yingqi Lv, Yingchao Dong, Tianze Wang, Jiayi Wu, Zhenxing Zhang, Xinrui Li, Ruikang Han, Fengxun Lin

**Affiliations:** School of Education and Psychology, University of Jinan, Jinan, China

**Keywords:** orderliness, disorderliness, construal level, psychological distance, IAT

## Abstract

People are innately capable of exploring and detecting orderliness and of attempting to make the world in which they live more orderly rather than more disorderly. Construal level theory asserts that the same stimuli can be represented abstractly or concretely and that psychological distance can affect the construal level. No research, however, has examined whether perceived orderliness/disorderliness is mentally associated with construal level and psychological distance. In this study, by using the Implicit Association Test (IAT), we conducted 10 studies to examine this possibility. The results of studies 1A–1B showed that people tended to associate high-level construal concepts with orderliness concepts and low-level construal concepts with disorderliness concepts. By contrast, the results of studies 2A–5B revealed that people associated psychologically proximal concepts with orderliness concepts and psychologically distal concepts with disorderliness concepts. These studies demonstrated that orderliness/disorderliness is associated with both construal level and psychological distance, but in opposite directions, suggesting that construal level and psychological distance may have distinct natures.

## Introduction

In nature and culture, order and disorder are present everywhere, and humans can avoid or eliminate neither order nor disorder (Koole and Van den Berg, 2005; Vohs et al., 2013). Evolutionarily, humans have evolved to prefer order, structure, and patterns over disorder, randomness, and chaos (Canfield and Haith, 1991; Huettel et al., 2002). For example, even 2- to 3-month-old infants can seek and detect consistent patterns that would empower them to easily predict their environment (Canfield and Haith, 1991). In most research, disorder refers to the lack of visible perceived order (a peaceful and safe state) and self-control (an act of maintaining). The visible perceived order includes social and physical cues (Skogan, 1986; Taylor and Shumaker, 1990). Visual social disorder usually refers to people who are loitering on the streets, drinking to excess, taking drugs, or engaging in dangerous behavior. Visual physical disorder refers to the appearance of the physical environment, such as places that are dirty with vandalism and graffiti, high levels of noise, and buildings that are in disrepair or abandoned. On the other end of the continuum, visual social and physical order include quiet, drug-free people, no people loitering, and buildings that are clean and in good repair (Ross and Jang, 2000). Recently, from a cognitive perspective, Kotabe (2014) proposed that the operationalization of disorder perception was a state in which things are in non-patterned and non-coherent positions, whereas order perception is the reverse state. Therefore, the core distinction in disorder/order perception is the degree of orderliness, regularity, pattern, and rationality. Various studies have revealed that disorderly environments encourage impulsive and disorderly behaviors such as rule-breaking and crime (Geis and Ross, 1998; Braga et al., 1999; Linares et al., 2001; Keizer et al., 2008; Vohs et al., 2013; Chae and Zhu, 2014; Bossuyt et al., 2016; Kotabe et al., 2016). Until now, little has been known about whether the inborn capacity of seeking orderliness and the ubiquitous order and disorder in the environment are affected and associated with people’s mental abstract representations of stimuli, including events and objects.

Individuals have the ability to represent the same stimulus (events or objects) in a comparatively concrete or abstract manner. Construal level theory (CLT), a purely cognitive orientation theory, divided this abstract/concrete representation of stimuli into two levels: low- and high-level construal (Trope and Liberman, 2003; Dhar and Kim, 2007). High-level construal refers to extracting the gist from available information; thus, representations are more abstract, coherent, integrative, structured, schematic, and decontextualized. In contrast, low-level construal usually refers to subordinate or incidental features of events and is relatively concrete, specific, disparate, unstructured, annalistic, and contextualized. For example, a hamburger could be highly construed as a tasty way to satisfy one’s hunger versus being construed at a low level as a combination of meat, vegetables, sugar, salt, and so on. In short, high-level construal is more structured and parsimonious but less rich and detailed than low-level construal (Trope and Liberman, 2003, 2010; Trope et al., 2007). The abovementioned definitions of disorder and low-level construal versus order and high-level construal imply that in daily life, the experience or perception of disorderliness might be construed at a low level, whereas the experience or perception of orderliness might be represented in a high-level manner. However, a more comprehensive experimental examination of the association between orderliness/disorderliness and high-/low-level construal is needed.

Construal level theory contends that psychological distance could affect construal level. Psychological distance usually refers to the perceiver’s set of subjective or direct experiences of the distance from a stimulus (Trope and Liberman, 2010). Psychological distance has opposing poles: proximal (e.g., caring for the aged, a star) or distal (e.g., dining, parent). Ample research shows that psychological distance influences how people think (Trope and Liberman, 2003, 2010) and how people feel (Leaf et al., 2010). By using an Implicit Association Test (IAT), Bar-Anan et al. (2006) demonstrated that participants automatically associate psychologically distal concepts with high-level construal and psychologically proximal concepts with low-level construals and that this process occurred without conscious deliberation. Furthermore, the four dimensions of psychological distance, i.e., spatial, temporal, social, and hypothetical, are interrelated (Yoav et al., 2007). Given the implicit association between construal level and psychological distance, it would be expected that psychological distance might also have some correspondence with the disorderliness/orderliness concept.

### Affective Reactions to Orderliness/Disorderliness and High-/Low-Level Construal

A series of articles have documented that construal level influences one’s affective reactions, such that construal level directly changes one’s affective valence of experiences. That is, experiences are more positive when represented in an abstract way than when represented in a concrete way (e.g., Eyal et al., 2004; Fujita et al., 2006; Williams et al., 2014). For example, Eyal et al. (2004) found that abstract/high-level construal of one’s experiences boosted focus on positive reasons or arguments (reasons supporting a course of action vs. reasons against a course of action). Fujita et al. (2006) found that abstract but not concrete thinking facilitated the pursuit of largely positive and desirable goals. Five experiments performed by Labroo and Patrick (2009) demonstrated that, compared with negative mood, a positive mood increased abstract construal, as reflected by the finding that positive smiling cues (vs. frowning) activated an abstract construal of activity; people in a positive mood preferred to construe actions more abstractly (i.e., the “why” aspect of an activity) than people in a negative mood; subjects in a positive mood valued academic goals as more important when primed by why they studied for exams (high-level construal condition), whereas subjects in a negative versus positive mood evaluated academic goals as more important when primed by how they studied for exams (low-level construal condition); and participants in a positive mood preferred products advertised with abstract framing. Recently, Williams et al. (2014) directly proposed and demonstrated that construal level could influence evaluation via shifting of affective valence; that is, experiences seemed more positive in an abstract manner than in a concrete manner, even if an experience was assumed to be negative and disliked. Through these studies, we are not attempting to summarize that abstract (high-level) construal always promotes positive experience or evaluation of events, but conversely, that concrete (low-level) construal enhances the negative experience or evaluation of events.

Various studies have revealed similar affective reactions that were induced by high-level construal and orderliness versus low-level construal and disorderliness. In contrast to ordered experiences or perceptions, varying lines of research have emphasized that disordered experiences or perceptions mostly result in negative affective consequences, including a powerlessness sensation (Geis and Ross, 1998), distress (Cutrona et al., 2000), feeling unsafe (Perkins et al., 2010), depression (Ross and Jang, 2000), and self-reported anxiety (Tullett et al., 2015). For example, using data from a representative sample of 2,482 adults (aged 18–92 years) in Illinois, Geis and Ross (1998), Ross (2000), and Ross and Jang (2000) found that compared with people who reported that they lived in an ordered neighborhood (e.g., quiet, clean), people who reported that they lived in a disordered neighborhood had significantly higher levels of feelings of powerlessness, depression, anxiety, malaise, and illness. Recently, Tullett et al. (2015) found that randomness (disorderliness) boosted anxiety when contrasted with orderliness, as reflected by larger error-related negativity (ERN) under disordered conditions. In short, much evidence has suggested that disorderliness elicits negative affect whereas orderliness evokes positive affect.

Given this empirical evidence that high-level construal and orderliness similarly induce positive affect and the connection of low-level construal and disorderliness with negative affect, it is not unreasonable to assume that there might be implicit connections between disorderliness and low-level construal versus orderliness and high-level construal.

### Processing Fluency/Disfluency Triggered by Orderliness/Disorderliness and High-/Low-Level Construal

Processing fluency is a meta-cognitive experience of the ease or difficulty of processing information (Oppenheimer, 2008) and has been associated with higher ratings of confidence in judgments, liking, truth, frequency, and willingness (see Alter and Oppenheimer (2009) for a review). Vallacher and Wegner (1987) demonstrated that feelings of ease are associated with high-level construal. However, a recent study directly and robustly demonstrated that cognitive fluency prompted people to process information more concretely, whereas disfluency boosted abstractly processing information (Alter and Oppenheimer, 2010). In the first experiment, by changing the font legibility of the questionnaire (perceptual fluency manipulation), Alter and Oppenheimer (2010) found that participants estimated cities as farther away when they received information about the cities in a questionnaire with a difficult-to-read font (disfluency condition) compared to where they received it from an easy-to-read-font questionnaire (fluency condition); furthermore, participants described New York City more abstractly when information was presented in a difficult-to-read font than when it was presented in an easy-to-read font. In another experiment, conceptual priming fluency was manipulated, and the results revealed that participants judged a city as closer and construed it more concretely when they were primed with its name first. In the third experiment, participants created more abstract definitions and descriptions for hard-to-pronounce obscure words and more concrete definitions for easy-to-pronounce obscure words. In short, fluently processed stimuli were perceived as more psychologically proximal than disfluently processed stimuli and further were rated as near and perceived as more concrete.

Because visually disordered stimuli include more information and seem more redundant than visually ordered stimuli, viewing visually disordered stimuli would be more cognitively demanding than viewing visually ordered stimuli at a high processing level (see Kinchla, 1977; Field, 1987; Olshausen and Field, 1996). Therefore, orderliness might be cognitively processed more fluently than disorderliness (Alter, 2013; Kotabe et al., 2016). Our recent ERP research found that the ERPs elicited by stimuli were less negative (more positive) in amplitude than those elicited by disordered stimuli at the frontal electrodes (represented by F7/F8, FT7/FT8, Fz, and FCz), whereas at the posterior electrodes (represented by P7/P8, PO7/PO8, Pz, and POz), the opposite was true, demonstrating that disordered stimuli were cognitively processed more disfluently than ordered stimuli (Li et al., 2019). From the cognitive fluency/disfluency processing viewpoint, order perception or experience might be linked with concrete (low-level) construal, whereas disorder perception or experience might be connected with abstract (high-level) construal.

In the present investigation, given these similarities between construal level and orderliness/disorderliness, i.e., the definitions, affective experiences, and triggered processing fluency/disfluency, we used the IAT (Greenwald et al., 1998, 2002) to assess the association between concepts of orderliness (e.g., clean and tidy, norm) vs. disorderliness (e.g., cluttered, strewn at random), construal level, and psychological distance in a straightforward manner. As introduced by Greenwald and colleagues, the IAT test was created to measure the free associations between a target and concept without being affected by elaborate thought or conscious preferences. The assumption is that a stronger association between the target and concept will result in a faster response. Therefore, the IAT may reflect the types of associations among orderliness/disorderliness, level of construal, and psychological distance that the present research aims to investigate.

Given that the definition of order is similar to that of high-level construal and order perception and high-level construal both trigger positive affect, and given that the definition of disorder is similar to that of low-level construal and disorder perception and low-level construal both elicit negative affect, we used pairings of orderly/disorderly words and words with different levels of construal that were either congruent with CLT or incongruent with CLT.

Hypothesis 1: People tend to associate orderly words with high-level construal words (categories or abstractness types) and disorderly words with low-level construal words (exemplars or concreteness types) (CLT-congruent pairings) more than people tend to associate orderly words with low-level construal words and disorderly words with high-level construal words (CLT-incongruent pairings).

Furthermore, past CLT research findings have proposed that abstract, high-level construal is linked to psychologically distant cues, whereas concrete, low-level construal is linked to psychologically proximal cues. Would similar results emerge across the four dimensions of psychological distance (temporal, spatial, social, and hypothetical)? We used pairings of orderly and disorderly words that were either congruent or incongruent with words of psychological distance (temporal, spatial, social and hypotheticality dimensions).

Hypothesis 2: People tend to associate orderly words with psychologically distal words (words on the psychologically distal pole or events/objects) and disorderly words with psychologically proximal words (words on the psychologically proximal pole or events/objects) (psychological distance (PD)-congruent pairings) more than people tend to associate orderliness with psychologically proximal words and disorderliness with psychologically distal words (PD-incongruent pairings).

However, because disorder perception and high-level construal both trigger cognitively disfluent experiences compared to order perception and low-level construal, which have both been shown to elicit cognitively fluent experiences, alternative hypotheses should exist.

Hypothesis 3: People tend to associate orderly words with low-level construal words and disorderly words with high-level construal words (defined as CLT-incongruent pairing based on hypothesis 1) more than people tend to associated orderly words with high-level construal words and disorderly words with low-level construal words (defined as CLT-congruent pairing based on hypothesis 1);

Hypothesis 4: People tend to associate orderly words with psychologically proximal words and disorderly words with psychologically distal words (defined as PD-incongruent pairing based on hypothesis 2) more than people tend to associate orderly words with psychologically distal words and disorderly words with psychologically proximal words (defined as PD-congruent pairing based on hypothesis 2).

## Study 1—Iat 1A-B: Implicit Associations Between Orderliness/Disorderliness and High-/Low-Level Construal

The first two studies tested the relation between order/disorder concepts and high- versus low-level construal concepts. In study 1A, the two construal level concepts were object terms labeled *exemplars* (representing low-level, concrete construal) versus *categories* (representing high-level, abstract construal). In study 1B, the two construal level concepts were descriptive terms for construal levels labeled *concrete* versus *abstract*. When the instructions were compatible with CLT pairings (CLT-congruent), one response key was mapped for either orderly concepts or high-level construal concepts, and the other response key was mapped for either disorderly concepts or low-level construal concepts (orderly concepts + categories in study 1A and orderly concepts + abstract condition in study 1B); conversely, when the instructions were not compatible with CLT pairings (CLT-incongruent), one response key was mapped to either orderly concepts or low-level construal concepts, and the other response key was mapped for either disorderly concept or high-level construal concepts (orderly concepts + exemplars in study 1A and orderly concepts + concrete condition in study 1B). Based on *hypothesis 1*, participants were expected to respond faster in the CLT-congruent condition than in the CLT-incongruent condition. However, based on *hypothesis 3*, participants were expected to respond faster in the CLT-incongruent condition than in the CLT-congruent condition.

### Method

#### Participants

All participants in the current study were native Chinese speakers. Forty-seven students (22 males; mean age = 19.09 ± 0.98 years) voluntarily participated in study 1A, and 37 students participated in study 1B (19 males; mean age = 19.11 ± 1.25 years). When a participant’s accuracy rate was more than 2.5 SDs below the average accuracy, all of that participant’s data were excluded. The data from the remaining 45 participants (20 males, mean age = 19.13 ± 0.99 years) in study 1A and 36 participants (16 males, mean age = 19.22 ± 1.37 years) in study 1B were used. All 10 experiments were approved by the Institutional Review Board of the School of Education and Psychology at the University of Jinan, and each participant gave informed written consent. There were neither sex effects nor order sequence effects in these and subsequent experiments.

#### Materials

Using Chinese word-frequency norms, the average frequencies of all words used in all 10 studies were matched. Examinations of word frequency, word strokes, and word valence yielded no systematic differences between words in each group, which was measured by 20 other participants before testing with the IATs.

The IAT (Greenwald et al., 1998) was used to assess the implicit association between orderliness/disorderliness and construal level. Each of the four concepts or categories—orderliness, disorderliness, high-level construal, and low-level construal—included eight items (Chinese words) that were chosen as the stimuli. The orderliness category included eight words like symmetry and norm. The disorderliness category included eight words like cluttered and chaos; see Table 1 for the Chinese words and their English translations. As suggested by Bar-Anan et al. (2006), high-level construal and low-level construal were represented by eight “categories” (e.g., vegetables) and eight “exemplars” (e.g., Chinese cabbage) in study 1A, and there were eight words that denoted abstractness (e.g., general) and eight words that denoted concreteness (e.g., special) in study 1B. See Table 2 for the Chinese words and their English translations.

**TABLE 1:** Stimuli used for orderliness and disorderliness in the IAT studies.

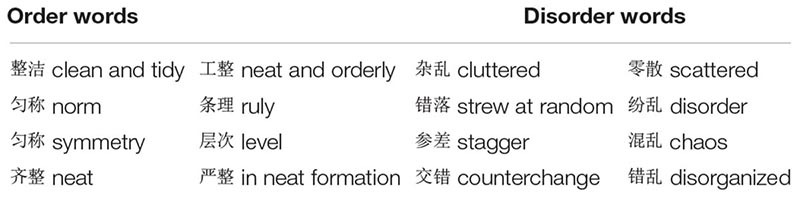	

**TABLE 2:** Stimuli used for construal levels in studies 1A–1B.

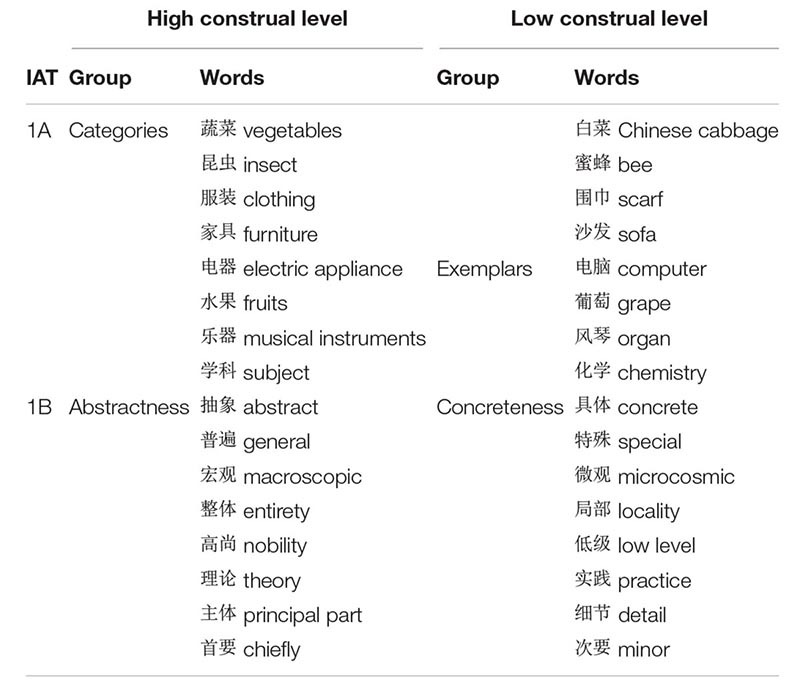	

#### Apparatus

Participants sat approximately 65 cm from a computer screen (a 22-inch Philips monitor, 1024 × 768 pixels, 85 Hz) attached to a Think Center M8200t computer and were asked to keep their heads on a head-rest with their eyes focused on the center of the screen during the test session. Stimuli were presented via E-prime 2.0 software (Psychology Software Tools, Inc., 2007) and accessed through laboratory computers. Responses were collected through the computer keyboard. Participants made left responses with the left forefinger (using the F key) and right responses with the right forefinger (using the K key).

#### Design

The IAT followed the standard blocks of categorization trials outlined by Greenwald et al. (1998). In study 1A, Block 1 consisted of 16 trials with orderliness/disorderliness items; Block 2 consisted of 16 trials with category/exemplar items (concrete/abstract items in study 1B); Block 3 was a combined practice block of 32 trials (the same label positions as in Blocks 1 and 2); Block 4 was a combined data collection block of 64 trials (the same label positions as in practice Block 3); Block 5 consisted of 16 category/exemplar items trials (with labels in the reversed positions from Block 2, concrete/abstract items in study 1B); Block 6 was a combined practice block of 32 trials (representing the new positions of category/exemplar—the same label positions as in Blocks 1 and 5); and Block 7 was a combined data collection block of 64 trials (the same label positions as in practice Block 6). The order of pairings was counterbalanced in these and all subsequent experiments. For instance, in experiment 1A, half of the participants completed an IAT with orderliness words and category items sharing a key in the first combined block, and half of the participants completed an IAT with orderliness words and exemplar items sharing a key in the first combined block. The sequence order of administration of congruent pairings (orderliness + high-level construal; disorderliness + low-level construal) and incongruent pairings (orderliness + low-level construal; disorderliness + high-level construal) was counterbalanced across participants.

#### Procedure

**FIGURE 1:**
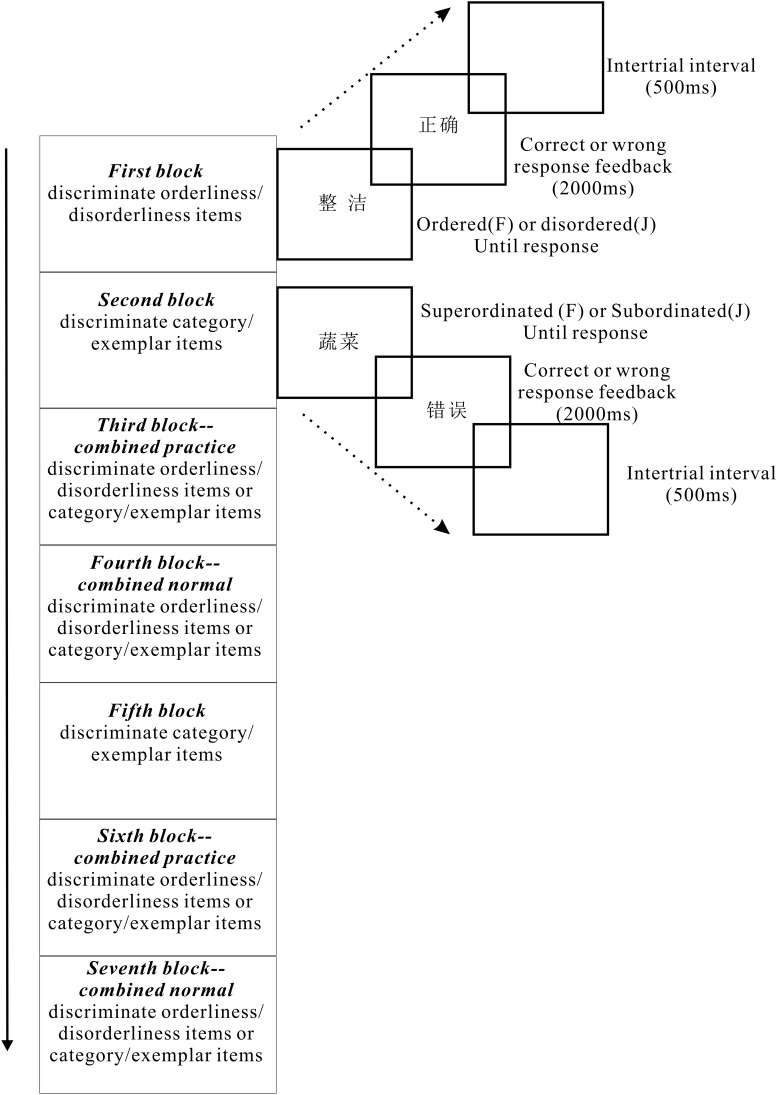
Illustration of the procedure for experiment 1A.

Each trial block started with instructions that described the category discrimination(s) for the block. The word was “ordered or disordered” or the item was “superordinate or subordinate” in study 1A, and the word was “abstract or concrete” in study 1B. The response keys “F” (left) and “J”(right) were for the discrimination. Words were presented in black against a white background and remained on the screen until the participant responded. The intertrial interval was 500 ms. A 2000-ms feedback was given when participants responded incorrectly, except for in Block 4 and Block 7, followed by the reappearance of the instructions written in red until participants pressed the space bar to continue. The procedure for 1A is illustrated in Figure 1. Words were presented randomly. The number of trials in each block ensured an equal number of appearances for all words.

### Results and Discussion

**FIGURE 2:**
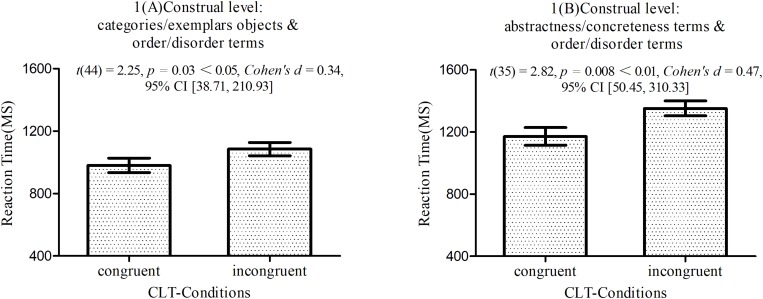
Response times for congruent and incongruent orderliness-construal level pairings for studies 1A and 1B. The differences between the conditions were significant in both experiments. Error bars represent 2 SEM.

This test uses reaction times in Block 4 and Block 7 to measure associations between concepts. Based on Greenwald et al. (1998), for extremely fast and extremely slow reactions, we recoded reaction times below 300 ms to 300 ms and those above 3000 ms to 3000 ms. As shown in Figure 2, in study 1A, performance was faster in the congruent condition (orderliness + categories and disorderliness + exemplars) than in the incongruent condition (disorderliness + categories and orderliness + exemplars) (*M*_incongruent_ = 1085.29 ms ± 285.19 versus *M*_congruent_ = 980.89 ms ± 311.07), *t*(44) = 2.25, *p* = 0.003 < 0.01, *Cohen’s d* = 0.34, 95% CI [38.71, 210.93], producing a significant IAT effect^1^. The same pattern of results emerged in study 1B (*M*_incongruent_ = 1351.92 ms ± 289.02 versus *M*_congruent_ = 1171.53 ms ± 341.55), *t*(35) = 2.82, *p* = 0.008 < 0.01, *Cohen’s d* = 0.47, 95% CI [50.45, 310.33].

The results of both study 1A and study 1B supported *hypothesis 1*, that high-level construal concepts were associated with orderliness concepts more than with disorderliness concepts and that low-level construal concepts were associated with disorderliness concepts more than with orderliness concepts.

## Study 2—Iats 2A–5B: Implicit Association Between Orderliness/Disorderliness and Psychological Distance

Each pair of studies related one dimension of psychological distance to orderliness/disorderliness. In studies 2A and 2B, we examined temporal distance; in studies 3A and 3B, we examined spatial distance; in studies 4A and 4B, we examined social distance; and in studies 5A and 5B, we examined hypothetical distance. For the first experiment in each pair, we examined the association between concepts that explicitly described either the psychologically proximal pole or the psychologically distal pole (e.g., for “near in time” vs. “distant in time” for the temporal dimension), and the indicators of order and disorder concepts were the same as those used in study 1. For the second experiment in each pair, we examined the association between concepts representing proximal versus distal events or objects (e.g., “real creatures” vs. “imaginary creatures” for the hypothetical dimension), and the concepts representing order and disorder were the same as those in study 1. The Chinese stimuli (translated into English) used in each of the studies are presented in Table 3. When the instructions were compatible with PD pairings, one response key was mapped for either orderly concepts or psychologically distal concepts, and the other response key was mapped for either disorderly concepts or psychologically proximal concepts; conversely, when the instructions were not compatible with PD pairings, one response key was mapped to either orderly concepts or psychologically proximal concepts, and the other response key was mapped for either disorderly concept or psychologically distal concepts. Based on *hypothesis 2*, we expected participants to respond faster in the PD-congruent condition than in the PD-incongruent condition. However, based on *hypothesis 4*, in the four studies, we expected participants to respond faster in the PD-incongruent condition than in the PD-congruent condition.

**TABLE 3:** Stimuli used for psychological distance in studies IATs 2A–5B.

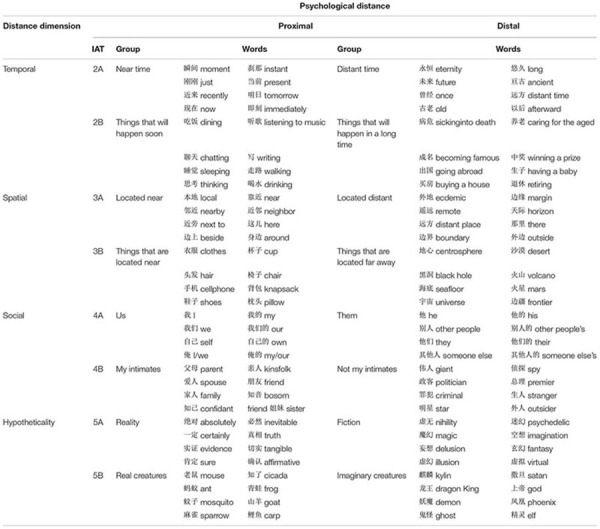	

### Method

#### Participants

Thirty-seven students (16 males; mean age = 19.33 ± 0.61 years) voluntarily participated in study 2A, 40 students participated in study 2B (21 males; mean age = 18.82 ± 0.79 years), 42 students participated in study 3A (22 males; mean age = 19.86 ± 0.68 years), 34 students participated in study 3B (12 males; mean age = 18.20 ± 0.74 years), 41 students participated in study 4A (21 males; mean age = 19.24 ± 1.43 years), 39 students participated in study 4B (17 males; mean age = 19.43 ± 1.57 years), 38 students participated in study 5A (18 males; mean age = 19.24 ± 1.85 years), and 39 students participated in study 5B (20 males; mean age = 19.31 ± 0.75 years). When a participant’s accuracy rate was more than 2.5 SDs below the average accuracy, all of that participant’s data were excluded. The data from the remaining 36 participants (10 males; mean age = 19.25 ± 0.60 years) in study 2A, 39 participants (20 males; mean age = 18.79 ± 0.77 years) in study 2B, 41 participants (19 males; mean age = 19.87 ± 0.68 years) in study 3A, 33 participants (12 males; mean age = 18.18 ± 0.68 years) in study 3B, 38 participants (18 males; mean age = 19.13 ± 1.45 years) in study 4A, 37 participants (17 males; mean age = 19.40 ± 1.46 years) in study 4B, 38 participants (18 males; mean age = 19.24 ± 1.85 years) in study 5A, and 37 participants (19 males; mean age = 19.27 ± 0.93 years) in study 5B were used.

#### Materials, Design, Apparatus, and Procedure

The IATs for studies 2A–5B used the same eight “orderliness” and eight “disorderliness” items as in the IAT for study 1A. The words representing psychological distance were all taken from Bar-Anan et al. (2006) (see Table 3 for Chinese words and their English translations).

The design was the same as in 1A, except that only the construal level or psychologically distant words were varied in each block.

The apparatus and procedure were also the same as in study 1A.

### Results and Discussion

**FIGURE 3:**
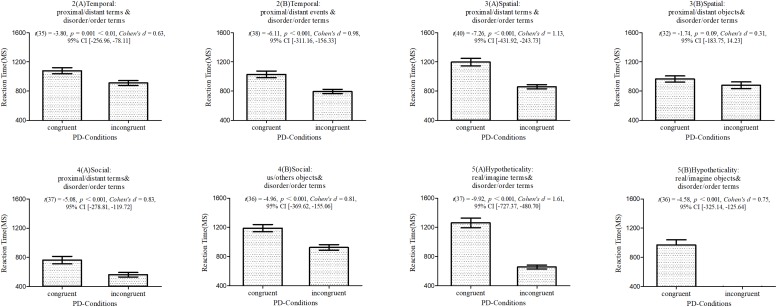
Response times for congruent and incongruent orderliness-distance pairings for studies 2A–5B. The differences between the conditions were significant in all experiments. Error bars represent 2 SEM.

In the seven IATs, responses were significantly (except for in 3B) faster in the incongruent condition (disorderliness + distance and orderliness + proximity) than in the congruent condition (orderliness + distance and disorderliness + proximity), all *t*s < −3.80, all *p*s < 0.05, and all *Cohen’s d* > 0.63 (the specific statistic values are shown in Figure 3). In experiment 3B, responses were marginally significantly faster in the incongruent condition than in the congruent condition, *t*(32) = −1.74, *p* = 0.09. The convergent results of studies 2A–5B showed that people tended to associate psychological proximity with orderliness and psychological distance with disorderliness more than psychological proximity with disorderliness and psychological distance with orderliness, and these findings verified *hypothesis 4.*

## General Discussion

In the current study, the IAT was used in 10 experiments to examine the associations between orderliness/disorderliness concepts and the concepts of construal level (low vs. high) and psychological distance (proximal vs. distal), which has four dimensions: temporal, spatial, social, and hypothetical. The results of studies 1A–1B provided support for ***hypothesis 1***, that people tended to associate orderliness concepts with concepts of high-level construal (categories or abstractness) and disorderliness concepts with concepts of low-level construal (exemplars or concreteness) more than they tended to associate orderliness concepts with concepts of low-level construal and disorderliness concepts with concepts of high-level construal. However, the convergent results of studies 2A–5B showed that people tended to associate psychological proximity concepts with orderliness concepts and psychological distance concepts with disorderliness concepts more than they tended to associate psychological proximity concepts with disorderliness concepts and psychological distance concepts with orderliness concepts, and these findings verified ***hypothesis 4***. Studies 2A, 3A, 4A, and 5A demonstrated that orderliness concepts were associated with indicators of proximity more than were disorderliness concepts. Experiments 2B, 3B, 4B, and 5B demonstrated that proximal object concepts were associated with orderliness concepts more than were distal object concepts. In sum, the current results revealed the implicit associations between orderliness/disorderliness and construal level versus orderliness/disorderliness and psychological distance, although the pattern of the association was opposite. The present research makes two important contributions. First, more generally, these studies demonstrate, for the first time, implicit conceptual associations between orderliness/disorderliness and construal level and psychological distance. Second, these findings further corroborate the previous proposition that although construal levels and psychological distance are related, they are not the same (Williams et al., 2014).

The results of studies 1A–1B revealed that people tended to associate high-level construal concepts with orderliness concepts and low-level construal concepts with disorderliness concepts. As mentioned in the introduction, construal levels directly alter the valence of affective experiences, such that when considered in an abstract manner rather than in a concrete manner, participants tended to experience more positively (Eyal et al., 2004; Fujita et al., 2006; Labroo and Patrick, 2009; Williams et al., 2014). Research has demonstrated that disorderliness evoked various negative effects, whereas orderliness elicited positive affect (Geis and Ross, 1998; Cutrona et al., 2000; Ross and Jang, 2000; Perkins et al., 2010; Tullett et al., 2015). Therefore, the conceptual association found in study 1 might result in the common positive affective experiences induced by both high-level construal and orderliness (positive) versus the negative affect experiences triggered by low-level construal and disorderliness. However, one unexamined issue should be noted. Similar valenced emotions may differ on other dimensions and then influence the construal level. For example, Chowdhry et al. (2015) recently found that disgust is a negative-valenced emotion, causing a relatively more abstract construal rather than a concrete construal. As we have mentioned, disorder perception or experience would elicit negative affect, including feelings of powerlessness (Geis and Ross, 1998), distress (Cutrona et al., 2000), lack of safety (Perkins et al., 2010), depression (Ross and Jang, 2000), and anxiety. Therefore, in the current study, we cannot distinguish which negative (vs. positive) affect linked by concrete construal (vs. abstract construal) resulted in the association with the disorder (order) concept. Future research should consider and examine this issue.

Research involving self-control could also explain the findings of studies 1A and 1B. Self-control refers to people failing to do what they want while having the knowledge, skill, and opportunity to do so (e.g., Kivetz and Simonson, 2002; Fujita et al., 2006). CLT has proposed that self-control was impacted by people’s subjective construal of events. For example, Fujita et al. (2006) manipulated abstract/concrete levels and assessed their effects on self-control and the underlying psychological processes. The results revealed and demonstrated that abstract construal promoted self-control success, whereas concrete construal tended to lead to self-control failure (Fujita et al., 2006; Fujita, 2010; Fujita and Roberts, 2010). A host of research articles examining disorderliness have supported the proposition that the sense of disorderliness or randomness was positively associated with the sense of failure or loss of self-control (Chae and Zhu, 2014; Kotabe, 2014) and that prolonged exposure to disorderliness may activate a mindset that things are random and uncontrollable, consequently reducing the motivation for self-control (see also Tullett et al., 2015). Therefore, it might be that the induced failure of self-control correlated the disorder concepts to the low-level construal.

However, as mentioned in the introduction, previous research results revealed that people were more likely to abstractly interpret the world when they experienced cognitive disfluency or difficulty than when they experienced cognitive fluency (Alter and Oppenheimer, 2010). If we were to adopt this processing fluency view, together with the aforementioned association of processing fluency with order perception and processing disfluency with disorder perception (Kotabe, 2014; Kotabe et al., 2016; Li et al., 2019), our study 1 should have found that people tended to associate orderly words with low-level construal words and disorderly words with high-level construal words. However, the results showed the opposite pattern: orderly words were more likely to be linked with high-level construal words than with low-level construal words, and the disorderly words were more likely to be connected with low-level construal words than with high-level construal words. In a review, Trope and Liberman (2010) stated that construal level refers primarily to the way in which people mentally represent information in an abstract or concrete manner, intrinsically focusing on the perception of *what* will occur, i.e., the processes that produce the representation of the event itself. It might be that abstract construal led to positive mood states and that positive mood rather than disfluency processing promoted the links between orderly words and high-level construal words.

Studies 2A–5B found that people tended to associate psychological proximity with orderliness and psychological distance with disorderliness more than they tended to associate psychological proximity with disorderliness and psychological distance with orderliness. This association pattern was contrary to the association pattern between construal level and orderliness/disorderliness. Williams et al. (2014) proposed and demonstrated that the effects of construal level and psychological distance on affect-based evaluations were distinct. Affective experience can be decomposed into valence (positive vs. negative) and intensity (the magnitude of the affective response) components (Russell, 1980; Russell et al., 1989). Psychological distance has an impact on the affect intensity rather than the affect valence, in which distance versus proximity could reduce the intensity of both the pleasure associated with positive experiences and the displeasure associated with negative experiences. In addition, as mentioned previously, disorder perception or experiences elicit negative affect, including depression (Ross and Jang, 2000) and anxiety (Tullett et al., 2015), and so on. However, anxiety and depression are differently associated with events in time: anxiety is associated with negative future events, and depression is linked with negative past events (Eysenck et al., 2006; Rinaldi et al., 2017). Therefore, the results of studies 2A–5B could not be explained in terms of affective valence.

One interpretation of the results of studies 2A–5B is from a cognitive processing fluency/disfluency perspective. Alter and Oppenheimer (2010) manipulated visual perceptual fluency, conceptual priming fluency, and linguistic fluency and found that stimuli that were processed fluently felt closer than those that were processed disfluently. Moreover, those fluently processing stimuli were judged as physically close. Therefore, processing fluency was identified as the underlying mechanism that motivates spontaneous inferences of psychological distance. Behavioral and ERPs studies in disorderliness and orderliness proposed and demonstrated that perceived disorder, compared to perceived order, was cognitively processed more disfluently (Kotabe, 2014; Kotabe et al., 2016; Li et al., 2019). Therefore, the disorderly words used in the current study might have triggered cognitive disfluency, and then this disfluency processing created a distant feeling for the disorderly words, whereas the orderly words had the opposite effect.

Another interpretation of studies 2A–5B should not be neglected. Psychological distance is an egocentric state because it arises from the subjective experience that something is close or far away from the self, here and now. Psychological distance mainly refers to perceiving events of *when, where, whom*, and *whether* it occurs, reflecting an automatic tendency of the mind (Trope and Liberman, 2003; Trope et al., 2007). Therefore, psychological distance from an event is more closely related to the spatiotemporal distance of the event from the self than to abstract or concrete representation. In addition, to some extent, the outcome of psychological distance follows directly from the biological relevance of physical distance (McGraw et al., 2012). Orderliness is usually comforting because of its association with predictability, and thus people are instinctively drawn to orderliness and can pursue goals confidently and interact with the environment effectively under conditions of orderliness (Harmonjones and Harmonjones, 2002). On the contrary, disorderliness can be undesirable because it prevents people from predicting what will happen next (Hirsh et al., 2012; Kotabe, 2014; Tullett et al., 2015). Inasmuch as there is an instinctive aversion to disorderliness, individuals would have a strong urge to recognize disorderliness as an event occurring in “the distant future” rather than in “the near future,” in “remote locations” rather than “nearby,” to “other people” rather than “oneself,” and as “unlikely” rather than “likely.” Therefore, the results in 2A–5B could verify this proposition, in which participants responded faster to distance-incongruent stimuli (in which disorderly words were linked with psychologically distal words and order words were linked with psychologically proximal words) than to distance-congruent stimuli (in which orderly words were linked with psychologically distal words and disorder words were linked with psychologically proximal words).

Previous research has focused on the degree to which psychological distance is implicitly associated with an abstract mindset and has the same effects regarding representation, prediction, evaluation, and behavior (e.g., Bar-Anan et al., 2006; Trope et al., 2007; Trope and Liberman, 2010). However, although there is a typical mutual correlation between psychological distance and construal level, this does not mean that the effects of abstraction manipulations will necessarily coincide with the effects of psychological distance manipulations on the same representation, prediction, evaluation, or behavior (Williams et al., 2014). Our findings are a reminder that the implicit association between particular constructs and psychological distance should not be interpreted as evidence that the particular construct and psychological distance shape outcomes via the same processes, since psychological distance and construal level were found to have an opposite association with orderliness and disorderliness in the current study. In conclusion, in the current study, we not only inventively demonstrated the implicit conceptual associations between orderliness/disorderliness, construal level, and psychological distance but also found evidence supporting a view that psychological distance and construal level are conceptually distinct, highlighting promising avenues for future research.

## Data Availability Statement

The raw data supporting the conclusions of this manuscript will be made available by the authors, without undue reservation, to any qualified researcher.

## Ethics Statement

All the ten experiments were approved by the Institutional Review Board of the School of Education and Psychology at the University of Jinan, and each participant signed the informed written consent.

## Author Contributions

KL, FL, and YL designed the experiment and wrote the manuscript. YD, TW, JW, and ZZ performed the experiment and analyzed the collecting data. YL, XL, and RH responded the reviewer comments, and YL also re-analyzed the collecting data and wrote the revised manuscript.

## Conflict of Interest

The authors declare that the research was conducted in the absence of any commercial or financial relationships that could be construed as a potential conflict of interest.

## Footnotes

1 Greenwald et al. (2002) also suggested an improved scoring measure for the IAT, called a D score, a variant of Cohen’s *d*. For each participant, a D score was computed by calculating the difference between the mean response times for the two double-categorization blocks (Blocks 4 and 7) within each participant’s IAT and dividing that difference by its associated pooled standard deviation. The statistical analysis revealed that the D scores of experiments 1A and IB were significantly higher than zero, *D*_1__A_ = 0.22,SD = 0.53, *t*(44) = 2.72, *p* = 0.009 < 0.01, 95% CI [0.056, 0.37]; *D*_1__B_ = 0.28, SD = 0.54, *t*(35) = 3.07, *p* = 0.004 < 0.01, 95% CI [0.094, 0.46], indicating a significant IAT effect. The D scores of experiments 2A–5B were all significantly lower than zero, *D*_2__A_ = −0.34, SD = 0.56, *t*(35) = −3.68, *p* = 0.001 < 0.01, 95% CI [−0.53, −0.15]; *D*_2__B_ = −0.55, SD = 0.46, *t*(38) = −7.45, *p* < 0.001, 95% CI [−0.70, −0.40]; *D*_3__A_ = −0.60, SD = 0.53, *t*(40) = −7.18, *p* < 0.001, 95% CI [−0.76, −0.43]; *D*_3__B_ = −0.25, SD = 0.56, *t*(32) = −2.62, *p* = 0.013 < 0.05, 95% CI [−0.44, −0.05]; *D*_4__A_ = −0.54, SD = 0.46, *t*(37) = −7.17, *p* < 0.001, 95% CI [−0.69, −0.39]; *D*_4__B_ = −0.47, SD = 0.52, *t*(36) = −5.49, *p* < 0.001, 95% CI [−0.64, −0.29]; *D*_5__A_ = −1.03, SD = 0.55, *t*(37) = −11.49, *p* < 0.001, 95% CI [−1.21, −0.84]; *D*_5__B_ = −0.50, SD = 0.53, *t*(36) = −5.71, *p* < 0.001, 95% CI [−0.68, −0.32], indicating a significant IAT effect
